# Flavonoid Functions in Plants and Their Interactions with Other Organisms

**DOI:** 10.3390/plants7020030

**Published:** 2018-04-03

**Authors:** Ulrike Mathesius

**Affiliations:** Division of Plant Science, Research School of Biology, The Australian National University, 134 Linnaeus Way, Canberra, ACT 2601, Australia; ulrike.mathesius@anu.edu.au

**Keywords:** biosynthesis, defence, herbivores, flavones, flavonoids, flower pigmentation, inflammation, lipid peroxidation, medicinal plants, nodulation, phenylphenalenones

## Abstract

Flavonoids are structurally diverse secondary metabolites in plants, with a multitude of functions. These span from functions in regulating plant development, pigmentation, and UV protection, to an array of roles in defence and signalling between plants and microorganisms. Because of their prevalence in the human diet, many flavonoids constitute important components of medicinal plants and are used in the control of inflammation and cancer prevention. Advances in the elucidation of flavonoid biosynthesis and its regulation have led to an increasing number of studies aimed at engineering the flavonoid pathway for enhancing nutritional value and plant defences against pathogens and herbivores, as well as modifying the feeding value of pastures. Many future opportunities await for the exploitation of this colourful pathway in crops, pastures, and medicinal plants.

Flavonoids are ubiquitous in plants. One of the interesting questions remains as to why plants produce such a variety of flavonoid metabolites—up to 10,000 across the plant kingdom. This special issue highlights some of the many functions that flavonoids have evolved to control plant development, plant–microbe interactions, and plant–animal interactions [1]. 

One of the very visible functions of flavonoids is in flower pigmentation, and, associated with that, in the attraction of pollinators and UV protection; this topic is exemplified by Dudek and colleagues, who explored the spatial occurrence of flavonoids in Papaver flowers, which present a beautiful model due to their wide-ranging variation in flower colour [2]. 

Our understanding of plant flavonoid synthesis and its regulation, especially in *Arabidopsis*, has seen major advances; these were explored by Zhang and Schrader, who present a stimulating and detailed review of the transcriptional control of the flavonoid pathway, how it has evolved, and what phenotypic consequences result from altered flavonoid biosynthesis in plants [3]. This topic is particularly relevant for strategies aimed at breeding or engineering of altered flavonoid content in crops, pastures, bioenergy plants, and in bacteria [4,5]. This is further explored by Nix and colleagues [6], who present a review on the flavonoid pathway in cotton, one of our most important crops. Of particular importance in cotton has been the research focused on the roles of flavonoids in cotton fibre production, as well as cotton defences against pathogens and herbivores. While the use of *Bt* cotton for improved pest management is widespread, future strategies utilizing flavonoids in pest management will be a fruitful avenue necessitated by the likely emergence of *Bt* resistance in pests. A further example of the utility of flavonoids and related metabolites, the phenylphenalenones, in the attack of bananas by weevils, their most important pest, is presented by Hölscher and colleagues [7], who identified a new compound involved in plant defence responses to weevil feeding. This will undoubtedly be important for breeding efforts to improve the pest resistance of banana.

One of the best-studied roles of flavonoids in plant–bacterial interactions is their structure-specific role in controlling nodulation gene expression [8,9] and infection of nitrogen-fixing rhizobia, which form a symbiosis with legumes. This topic was reviewed by Liu and Murray [10], who emphasised the intricate co-evolution of flavonoid production by host plants with their functions to control the specificity of symbiosis. The binding of flavonoids to transcriptional regulators inside rhizobia is necessary for their activation of downstream transcription of nodulation genes, but further roles of flavonoids in rhizobial infection and regulation of defence responses are less well understood at a mechanistic level and await future exploration. Seeing that flavonoid exudation by roots is common, and many bacteria use flavonoids as food sources [11], it appears fruitful to search for further targets for flavonoid action in the rhizosphere microbiome in the future. 

The insightful review of the flavone family by Jiang and colleagues paints a colourful picture of their biosynthesis and roles in plants, microbes, animals, and their interactions [12]. Not only have flavones taken on very specific functions in controlling plant development through their action in cell wall synthesis, but flavones also have activity in allelopathic action against other plants, in interactions with symbiotic mycorrhizal fungi and rhizobia, and in defence against fungal pathogens. An important aspect of flavone bioactivity is their structural diversity, which has allowed flavones to interact with a large variety of other molecules, determining their functions in lipid oxidation, DNA, and protein binding. These interactions underlie their multiple effects on human health, where their functions in the control of redox status, enzyme function, lipid peroxidation, inflammation, cell cycle, and cancer are well established. 

This is further explored in the articles by Navarro and colleagues [13] as well as Douros and colleagues [14], who demonstrate the broad roles of flavonoids in plants that have been used traditionally as medicinal plants like *Phyllanthus acuminatus* and *Cedrus brevifolia*, respectively. The structure–function relationships of known flavonoids are addressed in the article by Gutiérrez-Grijalva and colleagues [15], who demonstrated an array of function of flavonoids extracted from the oregano group of medicinal plants, and their dependency on specific modifications of the flavonoid backbone. It will be interesting to explore to what extent the action of flavonoids on animal enzymes has evolved from similar functions inside plants, a topic less studied.

## References

[1] Winkel-Shirley B. (2001). Flavonoid biosynthesis. A colorful model for genetics, biochemistry, cell biology, and biotechnology. Plant Physiol..

[2] Dudek B., Warskulat A.-C., Schneider B. (2016). The occurrence of flavonoids and related compounds in flower sections of *Papaver nudicaule*. Plants.

[3] Zhang B., Schrader A. (2017). TRANSPARENT TESTA GLABRA 1-dependent regulation of flavonoid biosynthesis. Plants.

[4] Dixon R.A., Steele C.L. (1999). Flavonoids and isoflavonoids—A gold mine for metabolic engineering. Trends Plant Sci..

[5] Falcone-Ferreyra M.L., Rius S.P., Casati P. (2012). Flavonoids: Biosynthesis, biological functions, and biotechnological applications. Front. Plant Sci..

[6] Nix A., Paull C., Colgrave M. (2017). Flavonoid profile of the cotton plant. *Gossypium hirsutum*: A Review. Plants.

[7] Hölscher D., Buerkert A., Schneider B. (2016). Phenylphenalenones accumulate in plant tissues of two banana cultivars in response to herbivory by the banana weevil and banana stem weevil. Plants.

[8] Liu C.-W., Murray J.D. (2016). The role of flavonoids in nodulation host-range specificity: An update. Plants.

[9] Peters N.K., Frost J.W., Long S.R. (1986). The flavone, luteolin, induces expression of *Rhizobium meliloti* nodulation genes. Science.

[10] Redmond J.R., Batley M., Djordjevic M.A., Innes R.W., Keumpel P.L., Rolfe B.G. (1986). Flavones induce expression of nodulation genes in *Rhizobium*. Nature.

[11] Cesco S., Neumann G., Tomasi N., Pinton R., Weisskopf L. (2010). Release of plant-borne flavonoids into the rhizosphere and their role in plant nutrition. Plant Soil.

[12] Jiang N., Doseff A.I., Grotewold E. (2016). Flavones: From biosynthesis to health benefits. Plants.

[13] Navarro M., Moreira I., Arnaez E., Quesada S., Azofeifa G., Vargas F., Alvarado D., Chen P. (2017). Flavonoids and ellagitannins characterization, antioxidant and cytotoxic activities of *Phyllanthus acuminatus* Vahl. Plants.

[14] Douros A., Hadjipavlou-Litina D., Nikolaou K., Skaltsa H. (2018). The occurrence of flavonoids and related compounds in *Cedrus brevifolia* A. Henry ex Elwes & A. Henry needles. Inhibitory potencies on lipoxygenase, linoleic acid lipid peroxidation and antioxidant activity. Plants.

[15] Gutiérrez-Grijalva E.P., Picos-Salas M.A., Leyva-López N., Criollo-Mendoza M.S., Vazquez-Olivo G., Heredia J.B. (2018). Flavonoids and phenolic acids from oregano: Occurrence, biological activity and health benefits. Plants.

